# The Four-Herb Chinese Medicine Formula Tuo-Li-Xiao-Du-San Accelerates Cutaneous Wound Healing in Streptozotocin-Induced Diabetic Rats through Reducing Inflammation and Increasing Angiogenesis

**DOI:** 10.1155/2016/5639129

**Published:** 2015-12-29

**Authors:** Xiao-na Zhang, Ze-jun Ma, Ying Wang, Yu-zhu Li, Bei Sun, Xin Guo, Cong-qing Pan, Li-ming Chen

**Affiliations:** 2011 Collaborative Innovation Center of Tianjin for Medical Epigenetics, Key Laboratory of Hormone and Development of Ministry of Health, Metabolic Disease Hospital and Tianjin Institute of Endocrinology, Tianjin Medical University, Tianjin 300070, China

## Abstract

Impaired wound healing in diabetic patients is a serious complication that often leads to amputation or even death with limited effective treatments. Tuo-Li-Xiao-Du-San (TLXDS), a traditional Chinese medicine formula for refractory wounds, has been prescribed for nearly 400 years in China and shows good efficacy in promoting healing. In this study, we explored the effect of TLXDS on healing of diabetic wounds and investigated underlying mechanisms. Four weeks after intravenous injection of streptozotocin, two full-thickness excisional wounds were created with a 10 mm diameter sterile biopsy punch on the back of rats. The ethanol extract of TLXDS was given once daily by oral gavage. Wound area, histological change, inflammation, angiogenesis, and collagen synthesis were evaluated. TLXDS treatment significantly accelerated healing of diabetic rats and improved the healing quality. These effects were associated with reduced neutrophil infiltration and macrophage accumulation, enhanced angiogenesis, and increased collagen deposition. This study shows that TLXDS improves diabetes-impaired wound healing.

## 1. Introduction

Nonhealing wound is a hallmark of diabetes and the leading cause of nontraumatic lower extremity amputation. The lifetime risk of a person with diabetes developing a chronic foot ulcer could be as high as 25% [1]. However, the treatments for it are limited and the cost is high [2]. Therefore, developing effective and economical therapies for correcting impaired healing of diabetic wounds is an urgent clinical demand. Diabetes impairs wound healing through magnifying the inflammatory response, inhibiting angiogenesis, and decreasing extracellular matrix (ECM) deposition [3]. The ideal treatment relies on correcting the multiple deficits simultaneously through highly integrated therapeutic approaches. Traditional Chinese medicine (TCM) is characterized by the use of herbal formulas that are usually grouped by two or more medicinal herbs, which can effectively produce synergetic effects to be greater than the sum of the individual effects and reduce side effects, providing novel therapeutic strategies for diabetic ulcer. Studies showed that combining TCM with conventional treatments in diabetic wound management received better clinical outcome [4].

Tuo-Li-Xiao-Du-San (TLXDS) is a refined Chinese medicine formula consisting of four herbs: Danggui (*Radix Angelica sinensis*), Huangqi (*Radix Astragali*), Baizhi (*Angelica dahurica*), and Zaojiaoci (*thorns of Gleditsia sinensis*), in the ratio of 5 : 5 : 4 : 4 (15 g for the former two and 12 g for the latter two). It is derived from “orthodox manual of surgery” (“Wai Ke Zheng Zong” in Chinese) formulated by a famous TCM physician Shigong Chen in 1617 AD and has been used for the treatment of various refractory wounds, including pressure ulcer, venous leg ulcer, abscesses, and carbuncle. In Chinese medicine theory, TLXDS includes the therapeutic method of “TUO” represented by* Radix Angelica sinensis *and* Radix Astragali* and the therapeutic method of “TOU” represented by* Angelica dahurica *and thorns of* Gleditsia sinensis. *Therapeutic method of “TUO” means raising “Qi” (vital energy) and nourishing “Blood” (body circulation), while therapeutic method of “TOU” refers to cleansing wound environment and eliminating toxins. For the pharmacological action of each single herb in TLXDS,* Radix Astragali* is used as “Qi” invigorator [5] and* Angelica sinensis* is prescribed as blood circulation activator [6];* Angelica dahurica* is classified as a sweat-inducing drug able to counter harmful external influences on the skin, such as cold, heat, dampness, and dryness [7]; the thorns of* Gleditsia sinensis* have been used for the treatment of inflammatory diseases including swelling, suppuration, carbuncle, and skin diseases [8]. Hundreds of years of practice has proven the wound healing effect of TLXDS on various refractory wounds [9], and the pharmacological actions of herbs in TLXDS suggest it might be a potential remedy for diabetic wound. However, the effect of TLXDS on healing of diabetic wounds has not been explored before. The purpose of this study is to evaluate the efficacy of TLXDS on diabetic wound by using an excisional cutaneous wound model of streptozotocin-induced diabetic rats and also to clarify its active mechanism by immunohistochemical, qRT-PCR, and western blot analyses.

## 2. Materials and Methods

### 2.1. Preparation of Tuo-Li-Xiao-Du-San Ethanol Extract

Tuo-Li-Xiao-Du-San is comprised of four herbs,* Astragalus membranaceus*,* Angelica sinensis*,* Angelica dahurica*, and thorns of* Gleditsia sinensis*, in the ratio of 5 : 5 : 4 : 4 (15 g for the former two and 12 g for the latter two). The herbs were obtained from and authenticated by TASLY Pharmaceutical Group Co. Ltd. (Tianjin, China). The 70% ethanol extract of mixture of four herbs was prepared by the department of Pharmaceutical Sciences, Tianjin University of Traditional Chinese Medicine (Tianjin, China) using standardized procedure [10, 11]. Briefly, the crude herbs were powered and then extracted by 70% ethanol for three times. Gather the extracts and filter to remove the solid fragment. The solvents were removed by freeze-drying. The condensate was stored at –20°C. The extracts are freshly prepared by dissolving in sterile water to an appropriate concentration as herb solution for the later experiment.

### 2.2. Animals

Sprague-Dawley male rats (*n* = 120, 10 weeks old, SPF) weighing 290 ± 10 g were purchased from Beijing HFK Bioscience Co., Ltd. (Beijing, China). Rats were housed under pathogen-free conditions at the Chinese Academy of medical Sciences & Peking Union Medical College Institute of Biomedical Engineering Animal SPF facility (Tianjin, China). Rats were maintained at controlled temperature (22–25°C) and relative humidity (50%–60%) on a 12 h light-dark cycle and fed a commercial pellet diet with food and water available* ad libitum*. This study was approved by Experimental Animal Ethical Committee of Tianjin Medical University, and all procedures with animals complied with rules of the Guide for the Care and Use of Laboratory Animals of the National Institutes of Health as well as the guidelines of the Animal Welfare Act.

### 2.3. Induction of Diabetes

After overnight fasting, diabetes was induced by a single intravenous injection of STZ (Sigma-Aldrich, St. Louis, MO, USA) at a dose of 50 mg/kg body weight in 0.1 mol/L citrate-phosphate buffer, pH 4.5. Control rats were injected with citrate buffer alone. Blood glucose concentration was monitored using an Accu-Chek Aviva glucometer (Roche Diagnostics GmbH, Germany) from tail vein blood. Animals with random blood glucose levels ≥16.7 mmol/L for three consecutive tests were considered diabetic.

### 2.4. Wound Model and TLXDS Treatment

Wound-healing model of rats was induced as described before [12]. Four weeks after STZ injection, control and diabetic rats were anesthetized by intraperitoneal injection of sodium pentobarbital (30 mg/kg body weight). The dorsal hair of rats was shaved and two 10 mm diameter full thickness wounds were created with a sterile biopsy punch. Diabetic rats were randomly allotted to diabetic treated with TLXDS (DM + TLXDS group, *n* = 36) and diabetic without drug treatment (DM group, *n* = 36), while nondiabetic rats were placed in the normal control group (NC group, *n* = 10). Rats of these three groups were randomly allocated to four experimental end points (i.e., days 5, 8, 11, and 14, 7–10 animals for each end point). Rats in TLXDS group received TLXDS ethanol extract 1.1 mL/0.2 kg body weight once daily by oral gavage starting from day 0 to each end point, and rats in NC and DM group received 1.1 mL/0.2 kg body weight water once daily by oral gavage. The dose of each herb used in rats was 1.5 g/kg body weight for* Angelica sinensis* and* Astragalus membranaceus*, 1.2 g/kg body weight for* Angelica dahurica* and* Gleditsia sinensis* thorns, which was calculated according to the dose used in patients (0.25 g/kg and 0.2 g/kg, resp.). At each experimental end point, the animals were killed simultaneously by euthanasia.

### 2.5. Macroscopic Analysis

The ulcers were photographed every other day with a digital camera. The percentage of completely closed ulcers was calculated with the NIH Image J analyzer by tracing the wound margin and calculating pixel area. Wound closure was calculated as Percentage Closed = [(Area on Day 0 − Open Area on Final Day)/Area on Day 0] × 100.

### 2.6. Immunohistochemistry

The wounds, together with unwounded skin margins, were excised, fixed with 10% formaldehyde, and embedded with paraffin. The sections (4 *μ*m thick) were then deparaffinized and rehydrated. Antigen retrieval was performed at 95°C by microwave in 0.01 mol/L sodium citrate buffer (pH 6.0). Endogenous peroxidase activity was quenched by exposing to 3% H_2_O_2_. After blocking with 5% BSA in PBS, the sections were incubated with anti-CD68 (1 : 100, Thermo Fisher Scientific), anti-MPO (1 : 100, Thermo Fisher Scientific), anti-CD31 (1 : 200, Santa Cruz Biotechnology), anti-desmin (1 : 100, Thermo Fisher Scientific), and anti-collagen I (1 : 100, Thermo Fisher Scientific), respectively, followed by incubating with the corresponding HRP-conjugated secondary antibodies. The antigen-antibody complex was visualized with a Diaminobenzidine (DAB) kit. For evaluation of staining, the overview of the positive-signal density was scored semiquantitatively as 1 (absent), 2 (low), 3 (medium), 4 (strong), and 5 (very strong). The median of scores from three observers, who were blinded to the treatment, was used for comparisons.

### 2.7. Collagen Estimation (Hydroxyproline Content)

Wound tissues were analyzed for hydroxyproline content, which is basic constituent of collagen. The collagen composed of amino acid (hydroxyproline) is the major component of extracellular tissue, which gives strength and support. Breakdown of collagen liberates free hydroxyproline and its peptides. Measurement of hydroxyproline hence can be used as a biochemical marker for tissue collagen and an index for collagen turnover. Hydroxyproline content was analyzed using a hydroxyproline assay kit (Sigma-Aldrich, St. Louis, MO, USA) according to the manufacturer's instructions. Dilute 10 *µ*L of the 1 mg/mL hydroxyproline standard solution with 90 *µ*L of water to prepare a 0.1 mg/mL standard solution. Add 0, 2, 4, 6, 8, and 10 *µ*L of the 0.1 mg/mL hydroxyproline standard solution into a 96-well plate, generating 0 (blank), 0.2, 0.4, 0.6, 0.8, and 1.0 *µ*g/well standards. Homogenize 10 mg tissue in 100 *µ*L of water and transfer it to a 2.0 mL polypropylene tube. Add 100 *µ*L of concentrated hydrochloric acid (HCl, ~12 M), cap tightly and hydrolyze at 120°C for 3 hours. Transfer 10–50 *µ*L of supernatant to a 96 well plate. Place plates in a 60°C oven to dry samples. Add 100 *µ*L of the Chloramine T/Oxidation Buffer Mixture to each sample and standard well. Incubate at room temperature for 5 minutes. Add 100 *µ*L of the Diluted DMAB Reagent to each sample and standard well and incubate for 90 minutes at 60°C. Measure the absorbance at 560 nm (A560). The concentration of the sample was calculated as

(1) Concentration  of  the  sample=OD  of  the  sampleOD  of  standard×Concentration  of  standard.

### 2.8. Real-Time Quantitative PCR

Total RNA from rat ulcer was extracted using Trizol (Invitrogen, Grand Island, NY). RNA purity and integrity were assessed by spectrophotometric analysis. A total of 3 *µ*g of RNA was reverse-transcribed using a RevertAid kit (Thermo Fisher Scientific, Waltham, MA). Reverse transcription polymerase chain reaction (RT-PCR) was performed using the CFX96 real-time PCR system (Bio-Rad, USA) with the SYBR Green PCR Kit (Takara, Otsu, Japan) for rat VEGF-A, PDGF-BB, IL-1*β*, and TNF-*α*. Primer sequences are as follows: for VEGF-A 5′ TCA AAC CTC ACC AAA GCC 3′ and 5′ GGT GAG AGG TCT AGT TCC 3′; for PDGF-BB 5′ CGC CTG CTG CAC AGA GAC 3′ and 5′ CCG CGA GAT CTG GAA CAC 3′; for IL-1*β* 5′ AGA AGA AGA TGG AAA AGC 3′ and 5′ CGA CCA TTG CTG TTT CCT 3′; for TNF-*α* 5′ TCC CAG GTT CTC TTC AAG G 3′ and 5′ GTA CAT GGG CTC ATA CCA G 3′; for GAPDH 5′ TAC CCA CGG CAA GTT CAA CG 3′ and 5′ CAC CAG CAT CAC CCC ATT TG 3′. GAPDH was defined as the reference gene. Data were analyzed with 2^−ΔΔCT^ method.

### 2.9. Western Blot Analysis

Protein contents of VEGF-A, PDGF-BB, IL-1*β*, and TNF-*α* in ulcer tissue homogenates were evaluated by western blot. In brief, the protein concentrations of homogenates were determined using a BCA protein assay (Pierce Biotechnology, Rockford, IL, USA). Equivalent amount of protein samples (30 *μ*g) was separated on an SDS polyacrylamide gel and transferred onto a polyvinylidene difluoride membrane (Millipore). After blocking in TBS containing 5% nonfat milk for 2 h at room temperature, the membranes were incubated overnight with 1 : 1,000 diluted anti-VEGF-A (Abcam), 1 : 2000 diluted anti-PDGF-BB (Abcam), 1 : 200 diluted anti-IL-1*β* (Santa Cruz Biotechnology), 1 : 500 diluted anti-TNF-*α* (Abcam), and 1 : 8000 diluted anti-*β*-actin (Tianjin Sungene Biotech Co., Ltd.), respectively. Binding of the primary antibody was detected using a HRP-conjugated secondary antibody (Tianjin Sungene Biotech Co., Ltd.). Positive bands were visualized using an ECL kit (Bio-Rad) and then captured on X-ray film. Housekeeping protein *β*-actin was used as a loading control. The density of each band was quantified using Quantity One software (Bio-Rad Laboratory) and normalized to their respective control.

### 2.10. Statistical Analysis

Data are presented as means ± SEM. Statistical analysis were performed using SPSS 16.0 software. One-way analysis of variance (ANOVA) test was used to determine statistical significance. *P* < 0.05 was considered to indicate a statistically significant difference.

## 3. Results

### 3.1. Diabetes Induction and Blood Glucose Level

**Figure 1:**
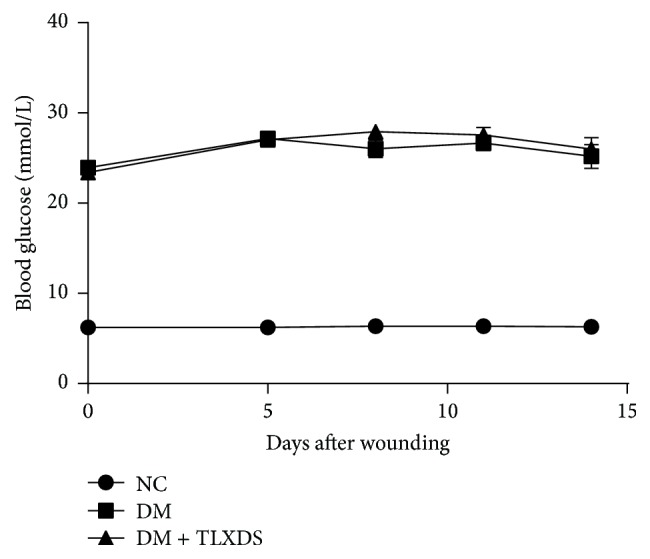
Blood glucose levels monitored during the treatment period. During the study period, the level of blood glucose was significantly higher in both DM and TLXDS-treated rats compared with the NC rats (*P* < 0.05). No significant difference between DM and TLXDS-treated groups was seen (*P* > 0.05). On day 0, *n* = 40 for NC, *n* = 36 for DM and DM + TLXDS; on day 5, *n* = 39 for NC, *n* = 34 for DM, and *n* = 35 for DM + TLXDS; on day 8, *n* = 29 for NC, *n* = 23 for DM, and *n* = 26 for DM + TLXDS; on day 11, *n* = 19 for NC, *n* = 15 for DM, and *n* = 16 for DM + TLXDS; on day 14, *n* = 9 for NC, *n* = 7 for DM, and *n* = 8 for DM + TLXDS.

During the 4-week diabetes induction period, 90% (72/80) of the STZ-injected rats became consistently hyperglycemic and were included in this study. During the treatment period, blood glucose levels of rats in DM group and DM + TLXDS group were significantly higher than those of rats in NC group (*P* < 0.05), and no significant difference was observed between DM group and DM + TLXDS group (*P* > 0.05), indicating that TLXDS had no effects on blood glucose (Figure 1).

### 3.2. Administration of TLXDS Accelerated Wound Healing

**Figure 2:**
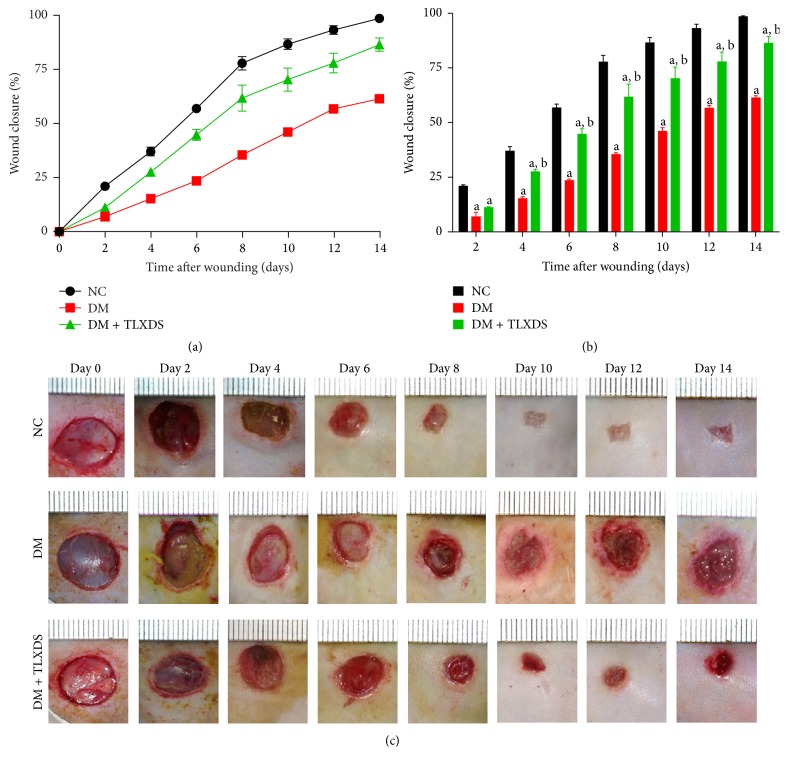
TLXDS treatment accelerated wound healing in diabetic rats. Percentage of wound closure (mean ± SEM) of 10 mm punch biopsies was monitored every other day until day 14 (a, b). Healing of diabetic wounds was significantly delayed compared with nondiabetic wounds at all observation time points (*P* < 0.05). TLXDS began to significantly improve wound closure on day 4 (*P* < 0.05). At the end of observation (14 days), 86.8% of the wounding area healed in DM + TLXDS group, while the closure rate in DM group was only 61.6%. (c) Typical photographs of wound healing for each group. ^a^ *P* < 0.05, compared with NC group, and ^b^ *P* < 0.05, compared with DM group. *n* = 7 for each group at each monitored time point.

As shown in Figure 2, by the end of observation (14 days after wounding), nondiabetic wounds completely healed, while most of the diabetic wounds remained open with a low average closure rate of 61.6%. TLXDS began to significantly improve diabetic wound closure 4 days after wounding (24% versus 14%, *P* < 0.05), and by the end of observation, TLXDS increased the healing rate of diabetic wound by 25.2% (86.8% versus 61.6%, *P* < 0.01).

### 3.3. Administration of TLXDS Reduced Inflammatory Cells Infiltration and Stimulated Inflammation Resolution

**Figure 3:**
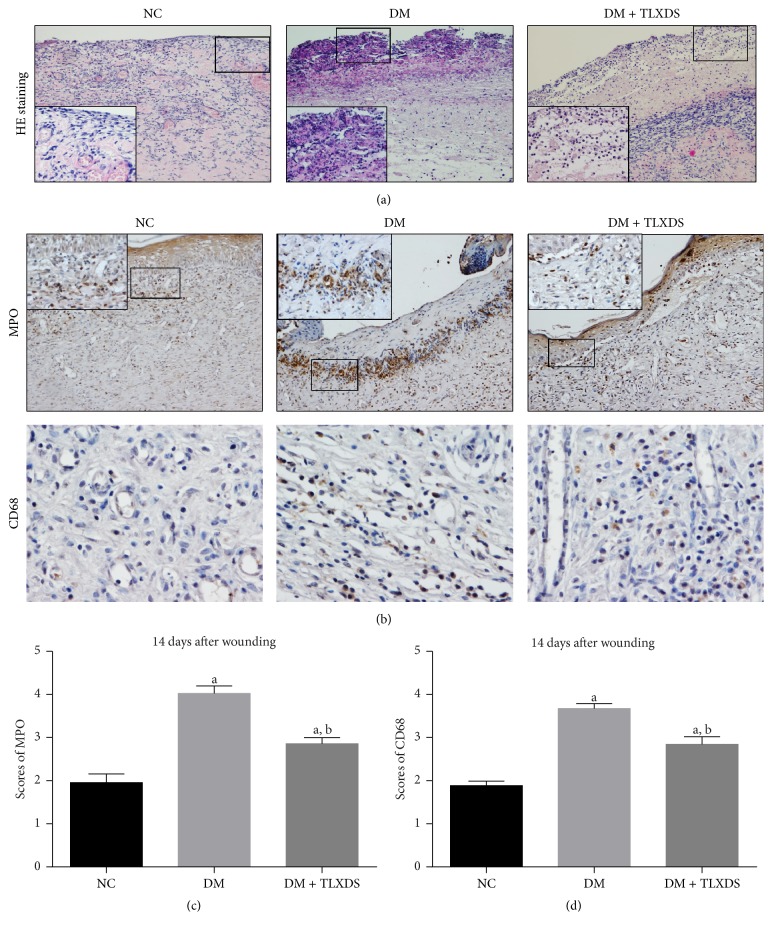
Effects of TLXDS treatment on inflammatory cells infiltration and inflammation resolution assessed by hematoxylin and eosin- (H&E-) stained histology on day 5 (a) and immunohistochemistry of MPO and CD68 on day 14 (b). Original magnification ×100 and insert magnification ×400. (c) and (d) Scores of MPO and CD68 staining. *n* = 7 for each group. Data are represented as means ± SEM. ^a^ *P* < 0.05, compared with NC group, and ^b^ *P* < 0.05, compared with DM group.

Uncontrolled inflammation is a major characteristic of diabetic wounds. We assessed histological changes by HE staining on day 5 (Figure 3(a)) and populations of neutrophil and macrophage at the wound site by determining the constitutively expressed molecular markers MPO (for neutrophils) and CD68 (for macrophages) on day 14 (Figures 3(b), 3(c), and 3(d)). The infiltration of inflammatory cells and concomitant tissue necrosis in diabetic wounds was much stronger compared with nondiabetic wounds on day 5 (Figure 3(a)). Moreover, the inflammation resolution of untreated diabetic rats was significantly delayed, which was characterized by increased and prolonged neutrophils and macrophages influx on day 14 (Figure 3(b)). Administration of TLXDS led to a marked decline in inflammatory cells infiltration and tissue necrosis on days 5 and an accelerated resolution of neutrophils and macrophages on day 14 (*P* < 0.05).

### 3.4. Administration of TLXDS Augmented Neovascularization and Increased Granulation Tissue Deposition

**Figure 4:**
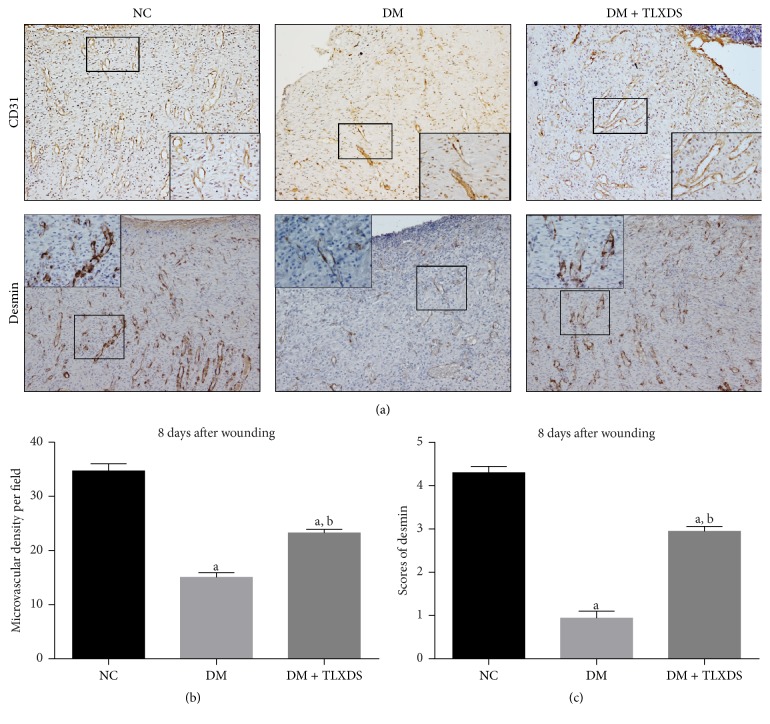
Effects of TLXDS treatment on angiogenesis assessed by the endothelial cell marker CD31 and pericyte marker desmin immunohistochemistry, respectively. (a) Representative CD31 and desmin-staining sections of NC group, DM group, and DM + TLXDS group on day 8 after wounding, respectively. Original magnification ×100 and inset magnification ×400. (b) Five “hot spots” in each specimen in which the CD31 antibody signal was the most intense were chosen and captured. The number of blood vessels was then counted by two investigators who were blinded to the treatment of the rats using the “manual tagging” feature in Image Pro-Plus software package. (c) Graphic visualization of scores of desmin staining on day 8. *n* = 7 for each group. Data are presented as means ± SEM. ^a^ *P* < 0.05, compared with NC group; ^b^ *P* < 0.05, compared with DM group.

Neovascularization is an essential event in the development of granulation tissue. We evaluated the growth and maturation of blood vessels by immunostaining of endothelial cell marker CD31 and pericyte marker desmin, respectively (Figure 4). The vessel density of untreated diabetic wounds was significantly decreased compared with nondiabetic wounds (*P* < 0.05), and the pericyte coverage of new vessels was discrete and incomplete. TLXDS treatment significantly enhanced neovascularization of diabetic wounds, demonstrated by increased CD31 staining. Besides, the pericyte recruitment was restored by TLXDS administration, suggesting that new vessels in TLXDS treated wounds were more mature, stable, and functional.

**Figure 5:**
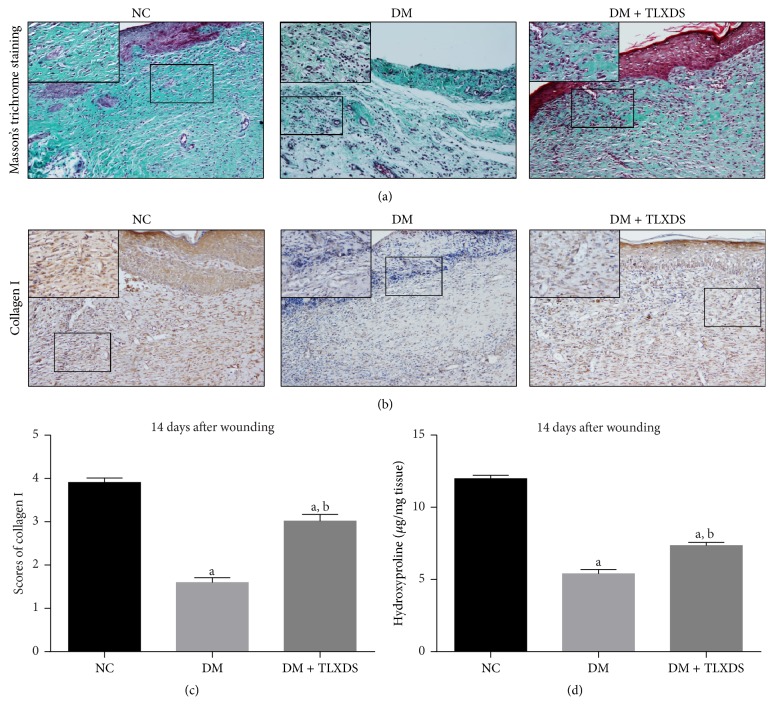
Effect of TLXDS treatment on extracellular matrix deposition evaluated by Masson's trichrome staining and collagen type I immunohistochemistry. Representative Masson's trichrome staining (a) and collagen type I staining sections (b) of NC, DM, and DM + TLXDS groups on day 14 after wounding. (c) Graphic visualization of scores of collagen type I staining on day 14. (d) Hydroxyproline content in the granulation tissue of each group on day 14. *n* = 7 for each group. Data are presented as means ± SEM. ^a^ *P* < 0.05, compared with NC group, and ^b^ *P* < 0.05, compared with DM group.

Meanwhile, Masson's trichrome staining and immunohistochemistry of type I collagen showed that the deposition of ECM in diabetic wounds on day 14 after wounding was significantly impaired compared with nondiabetic wounds, which was restored by the administration of TLXDS (Figure 5). Consistently, the hydroxyproline content in diabetic wounds was decreased significantly compared with nondiabetic wounds on day 14 (5.42 ± 0.29 versus 12.03 ± 0.24 *µ*g/mg tissue, *P* < 0.05). Administration of TLXDS increased hydroxyproline content in diabetic ulcers on day 14 (7.38 ± 0.22 versus 5.42 ± 0.29 *µ*g/mg tissue, *P* < 0.05).

### 3.5. Administration of TLXDS Increased Expression of Angiogenic Factors and Reduced Inflammatory Cytokine Expression

**Figure 6:**
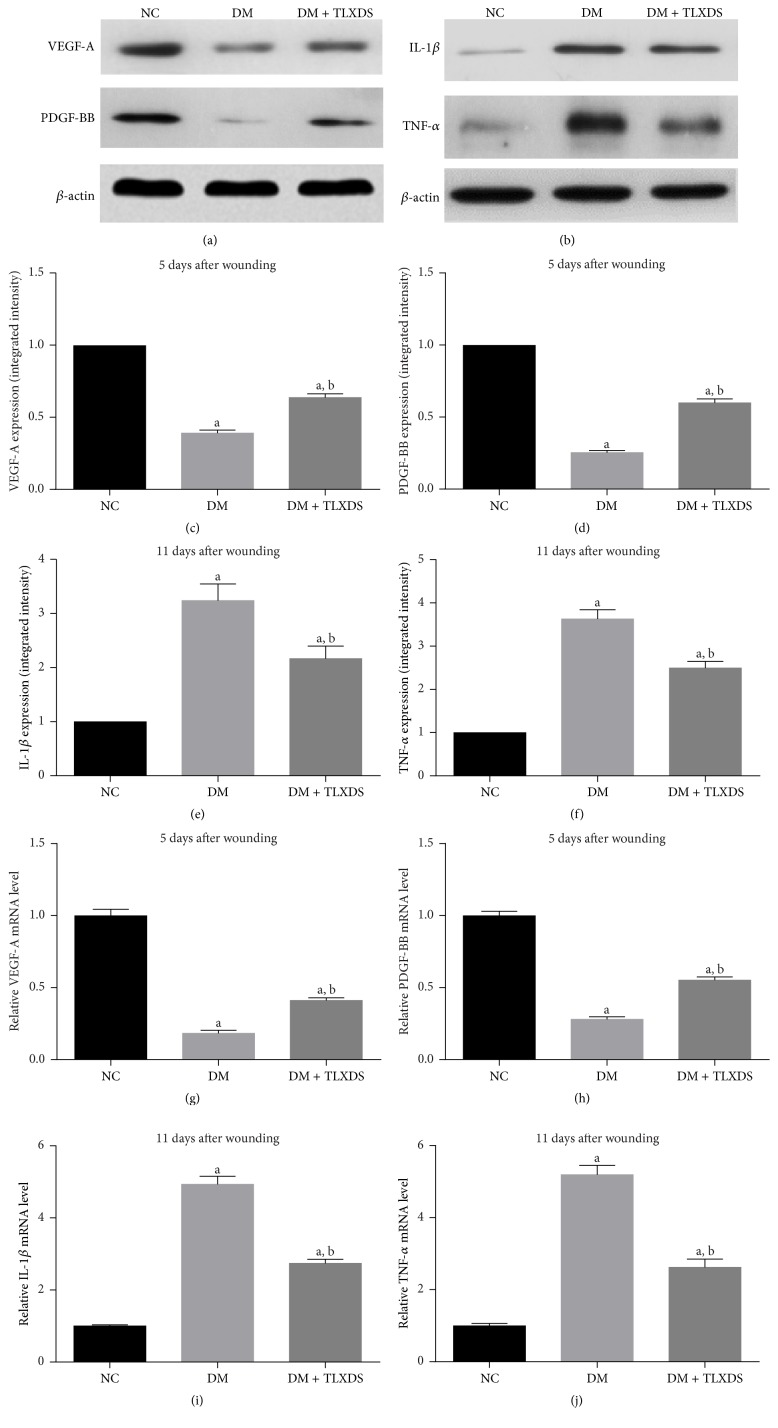
Effect of TLXDS treatment on the vascular endothelial growth factor-A (VEGF-A), platelet-derived growth factor BB (PDGF-BB), interleukin-1*β* (IL-1-*β*), and tumor necrosis factor-*α* (TNF-*α*) expressions assessed by western blot and real-time PCR analysis. Representative immunoblots of VEGF and PDGF-BB on day 5 after wounding ((a), *n* = 9 for each group), IL-1-*β* and TNF-*α* on day 11 after wounding ((b), *n* = 7 for each group). Quantifications of the bands (c–f). Quantification of VEGF-A, PDGF-BB, IL-1-*β*, and TNF-*α* mRNA expression (g–j). Data are presented as means ± SEM. ^a^ *P* < 0.05, compared with NC group, and ^b^ *P* < 0.05, compared with DM group.

Impaired angiogenic factors production accounts for the compromised neovascularization of diabetic wounds. Therefore, we performed real-time PCR and western blotting to investigate whether TLXDS increased neovascularization through increasing angiogenic factors expression. As expected, VEGF-A and PDGF-BB levels of diabetic wounds failed to rise on day 5 after wounding to initiate the angiogenic response as compared with nondiabetic wounds (Figures 6(a), 6(c), 6(d), 6(g), and 6(h)). TLXDS increased VEGF-A and PDGF-BB expression on day 5 after wounding when active angiogenesis was undergoing (*P* < 0.05).

TLXDS stimulated inflammation resolution in diabetic wounds. Therefore, we examined the effect of TLXDS on inflammatory cytokines expression on both gene and protein levels. On day 11 after wounding, expression of inflammatory cytokines, such as IL-1*β* and TNF-*α*, was significantly higher in diabetic wounds than in nondiabetic wounds (*P* < 0.05), and TLXDS markedly reduced these inflammatory cytokine expression (Figures 6(b), 6(e), 6(f), 6(i), and 6(j)).

## 4. Discussion

Impaired wound healing is a serious complication in diabetes, leading to prolonged hospitalization and even amputation. However, there are limited effective and safe treatments. In this study, we demonstrated that a traditional Chinese medicine formula for refractory ulcers, named Tuo-Li-Xiao-Du-San (TLXDS), significantly improved wound healing of STZ-induced diabetic rats through reducing inflammation, increasing angiogenesis and collagen deposition.

Wounds go through three sequential and coordinate phases, inflammation, tissue formation, and tissue remodeling, to restore morphological and functional integrity [13]. Diabetes impairs wound healing through magnifying the inflammatory response, inhibiting angiogenesis, and decreasing ECM deposition [3]. Inflammation is the first and indispensable response after acute skin injury, usually subsiding in less than 5 days after wounding [14]. However, metabolic defects, such as hyperglycemia and oxidative stress, induce excessive proinflammatory cytokines (IL-1*β*, TNF-*α*, etc.) production [15, 16], sustaining a prolonged influx of neutrophils and macrophages [14, 17, 18]. As shown in the present study (Figure 3), diabetic wounds were still stuck in large amount inflammatory cells infiltration and apparent tissue necrosis on day 5 after wounding, while nondiabetic wounds already moved forward to the proliferative stage of healing. Moreover, by 14 days after wounding, neutrophils and macrophages were still abundant in diabetic wounds. Persisting inflammatory cells create a protease (neutrophil elastase, MMPs, and gelatinase) rich hostile microenvironment [19], resulting in extracellular matrix and growth factors degradation [20, 21]. Therefore, diabetic wounds are entrapped in a self-sustaining cycle of chronic inflammation and never get mature enough to move forward to the next stage of healing. The present study showed that administration of TLXDS significantly accelerated inflammation resolution by decreasing the expression of inflammatory cytokines, such as IL-1*β* and TNF-*α*, and thereby reducing the neutrophils and macrophages abundance in diabetic wounds. Reducing inflammation by TLXDS treatment might provide a favorable microenvironment for other repair cells to play their roles in healing the wounds.

In response to hypoxia after injury, endothelial cells and pericytes are recruited by angiogenic factors from existing vessels and proliferate to form new and functional blood vessels which provide the essential oxygen and blood supply for regenerating new tissues, a process known as neovascularization [22, 23]. Inadequate neovascularization is a cardinal feature of nonhealing diabetic wounds. The mechanisms underlying this impairment are studied a lot and now researchers widely accept that inadequate production of angiogenic growth factors, such as VEGF and PDGF, is a fundamental cause [24–26]. In this study, we observed that TLXDS restores diabetes-impaired neovascularization by increasing recruitment of both endothelial cells and pericytes. The mechanisms of TLXDS's effects in boosting angiogenesis of diabetic wounds might be related, at least in part, to the correction of reduced production of angiogenic growth factors, such as VEGF-A and PDGF-BB, which is demonstrated in this study.

Furthermore, Masson's trichrome staining and collagen type I immunohistochemistry demonstrated that TLXDS treatment increased collagen production of diabetic wounds significantly, which is important for functional recovery and healing quality [27]. This effect is probably secondary to the combined action of reduced inflammation and increased angiogenesis, since the degradation of ECM was decreased and the synthesis was fueled.

Compound formulae, usually called “FuFang” in Chinese, are combinations of TCM prescribed for treating various diseases in China. The therapeutic potencies of herbs are found additive, or even synergistic, when used as combination. This is a great advantage for compound formulae in TCM. In Chinese medicine theory, TLXDS includes the therapeutic method of “TUO” which means raising “Qi” (vital energy) and nourishing “Blood” (body circulation) and the therapeutic method “TOU” which means cleansing wound environment and eliminating toxins. Recent researches show constituents of “TOU method,”* Angelica dahurica* and thorns of* Gleditsia sinensis*, exhibit antibacterial and anti-inflammatory activities [28, 29]; the constituents of “TUO method,”* Astragalus membranaceus* and* Angelica sinensis*, promote angiogenesis and the expression of angiogenic growth factors [30, 31]. Oftentimes, hundreds or even thousands of years of clinical practice has optimized formulae in TCM. The present study showed that TLXDS improves diabetic wounding healing as indicated in the ancient Chinese medicine theory and modern researching literature. There is great possibility that combining “TOU method” and “TUO method” can produce complementary and synergistic effects. It is of great interest in the future for us to reveal whether pleiotropic effects on diabetic wound healing of TLXDS in diabetic rats are attributed to the synergistic action between TUO method and TOU method. Furthermore, to clarify the active ingredients in TLXDS is of great practical significance as well.

It is noticeable that ulcer healing in diabetic ulcer and diabetic foot is complicated with many chronic problems, including long-term uncontrolled hyperglycemia, peripheral vascular disease, neuropathies, excessive pressure to the wound sites, and infection secondary to compromised immunity [2]. One pathogenic abnormality can lead to another, developing vicious cycles of pathogenicity in the diabetic chronic ulcers. Considering the heterogeneity and complexity of human diabetic foot, no single animal model is capable of fully recapitulating each clinical scenario [32]. In the present study, we employed a widely used STZ-induced diabetic animal wound model [33, 34] to simulate the hyperglycemic state of diabetics and its impairment on wound healing. The results showed that TLXDS treatment is able to correct the abnormal healing process induced by hyperglycemia through reducing inflammation, increasing angiogenesis, and collagen deposition. Whether TLXDS could improve other abnormalities leading to diabetic foot, such as vascular disease and neuropathies, will be a constructive investigative direction in our further research work by using appropriate animal or cell models. And whether these findings can be extrapolated to the situation encountered in diabetic foot in patients still needs further investigation in clinical trials.

In summary, this study showed that TLXDS, a traditional Chinese medicine formula for refractory ulcers, has a positive effect on diabetes-impaired wounds. The improved wound healing is associated with reduced inflammation, increased angiogenesis, and collagen deposition after TLXDS treatment. The oral administration of traditional Chinese herbal medicine could provide an alternative and effective approach for diabetic foot ulcer therapy.
